# Acute severe hyperphosphatemia and secondary hypocalcemia after phosphate enema administration in a constipated post-trauma patient recovering from pelvic surgery: a case report

**DOI:** 10.1093/jscr/rjag462

**Published:** 2026-06-13

**Authors:** Karoline B Bräuner, Hjalte L Berthelsen, Anne G Vedel

**Affiliations:** Department of Surgery, Slagelse Hospital, Fælledvej 11, Slagelse 4200, Denmark; Department of Internal Medicine, Slagelse Hospital, Fælledvej 11, Slagelse 4200, Denmark; Department of Anesthesia, Slagelse Hospital, Fælledvej 2, Slagelse 4200, Denmark

**Keywords:** hyperphosphatemia, dialysis, trauma surgery, emergency surgery

## Abstract

Severe hyperphosphatemia secondary to phosphate enema administration is a rare but clinically significant event, particularly in patients with renal impairment. We present a case of a 77-year-old male with a history of polytrauma and prolonged immobility, who developed severe hyperphosphatemia and secondary hypocalcemia following rectal administration of a phosphate enema. The case highlights hyperphosphatemia as a potential complication of phosphate absorption and underlines the importance of early recognition of symptoms and prompt treatment.

## Introduction

Severe hyperphosphatemia secondary to phosphate enema administration is a rare but clinically significant event [1–3], particularly in patients with renal impairment. It has, however, been presented in a few case reports and may contribute to an important differential diagnosis in patients with acute severe hyperphosphatemia or hypocalcemia as hypocalcemia frequently is seen secondarily to hyperphosphatemia due to bonding into crystals. This process may also constitute renal impairment [4, 5]. Therefore, the aim of this case report is to present a case of this rare complication for other clinicians to learn.

## Case report

**Figure 1:**
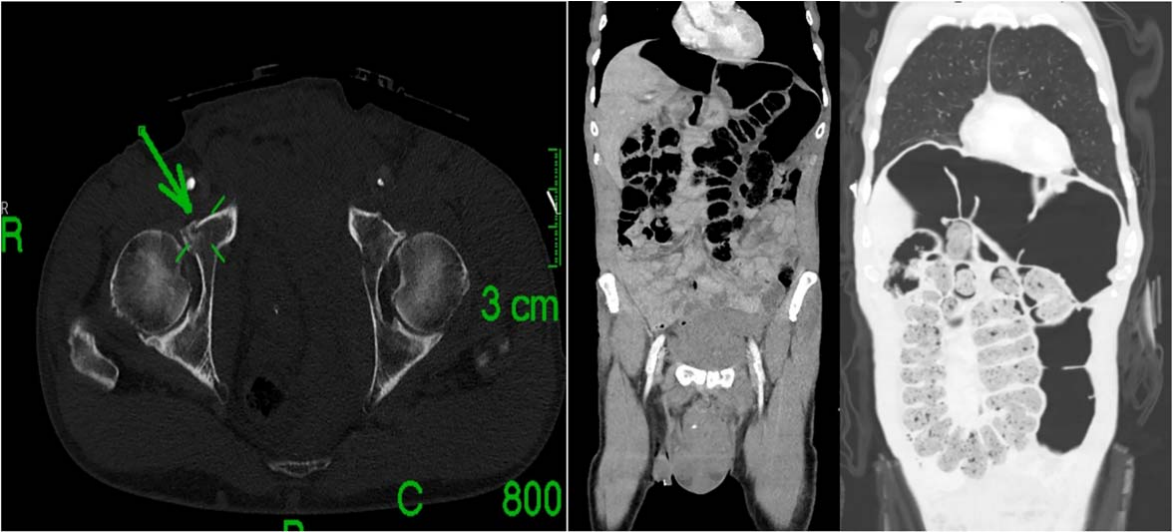
Trauma computed tomography (CT) of thorax and abdomen after initial trauma.

A previously healthy and active 77-year-old male sustained a complex pelvic fracture following a trauma that had him stuck under a 150-kilogram concrete block for approximately 90 minutes. The initial trauma computer assisted tomography (CAT) showed no acute bowel injuries but a complex pelvic fracture with bilateral ramus fracture (Fig. 1). Significant hematoma of the external genitals and left lower extremity was observed, and upon placement of a urinary catheter the patient presented with macroscopic hematuria. The patient was kept bedridden in the intensive care unit and underwent surgery in a tertiary care center (Odense University Hospital) four days following the trauma.

**Figure 2:**
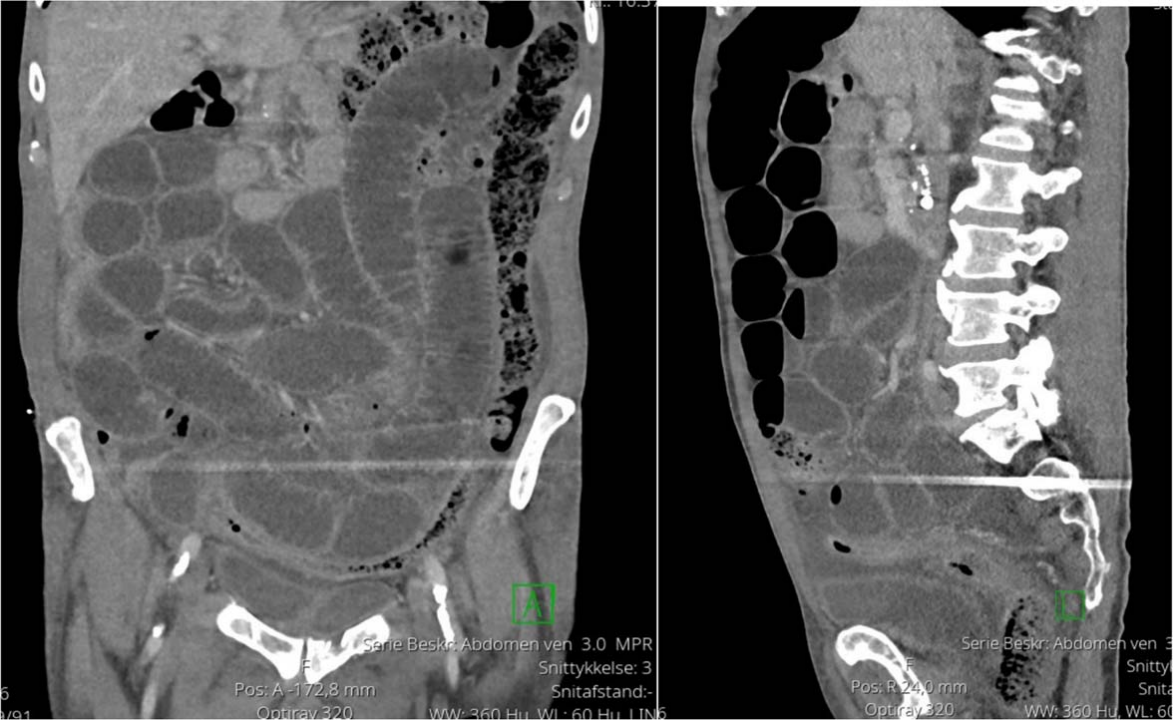
Abdominal surgery acute protocol (ASAP) CT scan of abdomen at first readmission on suspicion of intestinal obstruction.

The patient was readmitted four days after discharge with abdominal pain, nausea, and distension. A CAT scan revealed severe colonic constipation without definitive signs of obstruction and no free gas (Fig. 2). A phosphate enema (240 mL) was administered, after which the patient developed acute pain that subsided within 30 minutes.

Subsequent laboratory tests, performed to assess whether electrolyte derangement was a contributing factor in the development of constipation, showed an alarming phosphate level (>8.18 mmol/L) and a precipitous drop in ionized calcium (1.24 mmol/L to 0.75 mmol/L within six hours). Repeat testing confirmed these findings. The patient exhibited metabolic acidosis with an increased lactate level (1.1 mmol/L to 3.1 mmol/L) and clinical symptoms including severe muscle fatigue, tiredness, and dizziness. Nephrology and endocrinology consultations made via telephone suggested the high phosphate level explained by excessive phosphate absorption, likely exacerbated by mild renal impairment (creatinine 140 μmol/L at admission).

During the initial finding of severe hyperphosphatemia and hypocalcemia, the team considered possible causes for this: Undiagnosed parathyroid disorder, severe renal disease (unlikely due to near-normal creatinine) [6], tumor lysis of bone tumor (unlikely with no finding of malignancy in several recent CAT-scans) [7], leukemia (not likely with near-normal leucocyte counts) [8] or possibly related to the phosphate enema [1, 2], which seemed most likely.

The patient was transferred to the intensive care unit for close monitoring. Given the risk of calcium-phosphate precipitation in renal tubules [4, 5], intravenous calcium replacement was deferred initially, however briefly needed when ionized calcium levels became immeasurably low (< 0.5 mmol/L) and the patient accordingly symptomatic. Continuous renal replacement therapy (CRRT) was initiated, resulting in progressive normalization of phosphate and calcium levels within 48 hours [9, 10].

**Figure 3:**
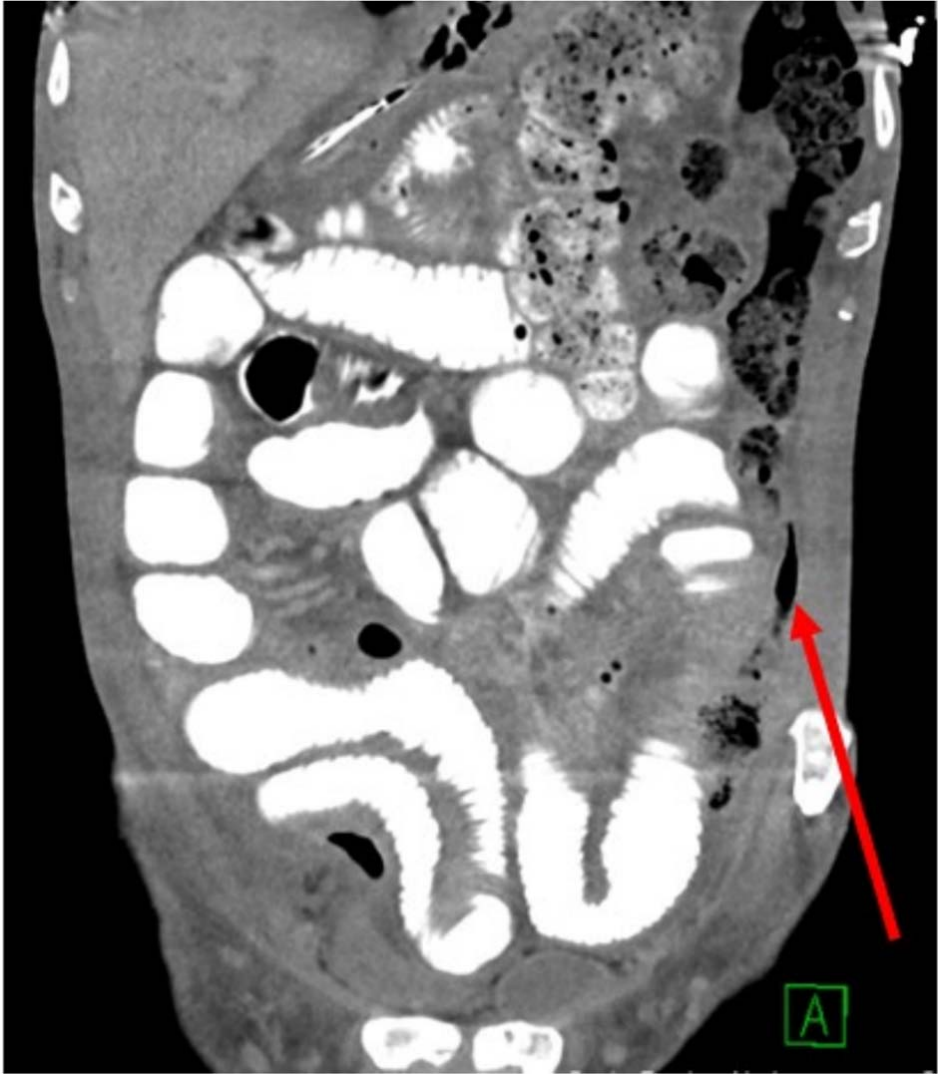
ASAP CT scan of abdomen after stabilization of electrolytes on suspicion of intestinal perforation.

Despite initial stabilization, the patient later developed peritonitis with radiologic evidence of sigmoid perforation [11] (Fig. 3). Diagnostic laparoscopy confirmed a longitudinal sigmoid perforation, likely related to increased intraluminal pressure. The defect was surgically repaired. It was later suspected that the sigmoid lesion was a primary trauma lesion later encapsulated by intraabdominal fat and therefore not visible on the CAT scan performed after readmission weeks after the initial trauma. The administration of the enema led to reperforation and thus enema spilled in the peritoneal cavity explaining both the patient’s breakthrough pain and severe hyperphosphatemia.

**Figure 4:**
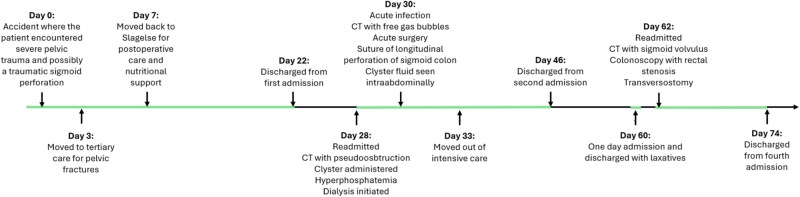
Overview of the entire treatment trajectory.

Postoperatively, the patient’s electrolyte levels remained stable, and he progressed well with rehabilitation (Fig. 4).

## Discussion

Phosphate enemas are widely used for bowel cleansing and biochemically their potential for hyperphosphatemia, particularly in patients with severely compromised renal function, is well described, especially in children [6]. Excessive phosphate absorption can lead to secondary hypocalcemia due to calcium-phosphate binding, which may promote calciphylaxis (the precipitation of calcium phosphate in tissues). Hyperphosphatemia *itself* is generally asymptomatic; however, the hypocalcemia may cause dizziness, muscle weakness, cramps, laryngospasms and altered mental status – symptoms that should prompt immediate evaluation of phosphate and calcium levels [10].

In hindsight, the hyperphosphatemia in this case was more severe than typically seen in patients suffering a light to moderate degree of acute kidney injury following phosphate enema administration. A fact later explained during surgery, where it was uncovered that the phosphate enema had found its way to the abdominal cavity allowing for peritoneal phosphate absorption rather than absorption from the bowel alone.

This case underscores the importance of cautious use of phosphate-based laxatives, especially in patients with renal impairment. Acute excessive hyperphosphatemia with secondary hypocalcemia should lead not only to intensive care consultation but also a critical approach to the etiology of the hyperphosphatemia, as accidental acute bowel perforation may be an explanation.

In conclusion, severe hyperphosphatemia is a rare clinical finding but should lead the clinician to thorough examination for the root cause, especially if developed rapidly. Additionally, since hyperphosphatemia in itself is not necessarily critical, measurements of ionized calcium should be taken to identify potentially life-threatening secondary hypocalcemia. Administration of intravenous calcium in these cases should be done cautiously to avoid calcium-phosphate precipitation in renal tubules. Acute dialysis can be necessary in the acute phase. Acute hyperphosphatemia in renal competent patients after administration of phosphate enemas should raise suspicion of rectal perforation.

## Key Takeaways

- Severe hyperphosphatemia is a rare finding and should prompt thorough clinical assessments to identify and treat the root cause.
- Symptoms such as dizziness and muscle fatigue post-enema should prompt evaluation for hyperphosphatemia and secondary hypocalcemia.
- Acute dialysis was an effective treatment in the acute phase, preventing further complications.
- Acute hyperphosphatemia or acute development of low ionized calcium levels after administration of phosphate enemas should raise suspicion of rectal perforation.
